# Extreme Risk Protection Orders and Firearm and Nonfirearm Suicides in the US

**DOI:** 10.1001/jamahealthforum.2025.6442

**Published:** 2026-01-30

**Authors:** Timothy T. Brown, Mark S. Kaplan, Zhimeng Yan, Yunyu Xiao

**Affiliations:** 1 School of Public Health, University of California, Berkeley; 2 Luskin School of Public Affairs, University of California, Los Angeles; 3 Department of Population Health Sciences, Weill Cornell Medicine | NewYork-Presbyterian, New York, New York

## Abstract

**Importance:**

Firearm suicides constitute a crisis in the US, accounting for more than half (55.4%) of all suicide deaths in 2023. Extreme Risk Protection Orders (ERPOs; ie, red flag laws) authorize temporary firearm removal from individuals deemed at high risk of harming themselves or others. While ERPOs are designed to reduce firearm-related suicides, whether they result in a net reduction in suicide deaths or shift firearm suicides to suicides by other methods remains an important but unresolved issue in determining their effectiveness.

**Objective:**

To determine the association of ERPOs with firearm suicides and nonfirearm suicides in states with sufficient postpolicy data and no confounding firearm legislation that may bias findings on ERPO outcomes.

**Design, Setting, and Participants:**

In this cohort study, 2-way difference-in-differences event study analyses were conducted using county-level data from 2012 to 2022. All states that passed ERPO laws alone, with no other new firearm laws, from 2018 to 2020, and had at least 1 year post–ERPO laws during which no new firearm laws were passed were investigated. All states that had no existing ERPO laws and passed no new firearm legislation from 2016 to 2022 were used for comparison. The model accounted for staggered treatment timing, treatment heterogeneity, and key methodological assumptions. Data were analyzed between February 6 and October 9, 2025.

**Exposure:**

State-level ERPO law passage.

**Main Outcomes and Measures:**

County-level annual firearm suicides and nonfirearm suicides per 100 000 population, derived from Centers for Disease Control and Prevention Wide-Ranging Online Data for Epidemiologic Research.

**Results:**

This study examined county-level data from 4 states passing ERPO laws alone (Massachusetts, New Jersey, New Mexico, and Rhode Island) compared with 8 that did not (Alabama, Alaska, Michigan, Minnesota, Nebraska, North Carolina, Pennsylvania, and South Carolina). ERPO passage was associated with a mean reduction of 3.79 firearm suicides per 100 000 population after 1 year (95% CI, −6.74 to −0.83; *P* = .01), equivalent to an estimated 675 suicides. With regard to nonfirearm suicides, no association was found in the year ERPO laws were passed (0.41; 95% CI, −1.21 to 1.94; *P* = .60) or in the next year (−2.45; 95% CI, −6.84 to 1.93; *P* = .27).

**Conclusions and Relevance:**

In this cohort study, ERPO laws in Massachusetts, New Jersey, New Mexico, and Rhode Island were associated with substantial reductions in firearm suicides, with no evidence of substitution with nonfirearm methods. These findings support ERPOs as targeted public health interventions to reduce firearm suicides without increasing suicides by other methods.

## Key Points

**Question:**

Were Extreme Risk Protection Order (ERPO) laws passed alone (no other firearm laws passed simultaneously or 1 year after) during 2018 to 2020 associated with fewer firearm suicides without shifts to nonfirearm suicides?

**Findings:**

In this cohort study of US county-level suicide data in 4 states with and 8 without ERPO laws, ERPOs were associated with 675 fewer estimated firearm suicides over the treatment period without measurable increases in nonfirearm suicides.

**Meaning:**

These results suggest ERPOs may serve as effective public health tools to reduce firearm suicides without increasing suicides by alternative methods.

## Teaser

This cohort study assessed the association between Extreme Risk Protection Orders and firearm suicide reductions in states passing no additional firearm laws.

## Introduction

Firearm suicides account for more than half of all suicide deaths in the US.^1^ Restricting access to lethal means, particularly firearms, is considered to be one of the most effective strategies to reduce suicide deaths.^2^ Extreme Risk Protection Orders (ERPOs), commonly known as red flag laws,^3,4^ authorize law enforcement officers and, in some cases, family and household members and clinicians to petition courts for temporary firearm removal from individuals deemed at high risk of harming themselves or others.^3,4^

Since Connecticut passed the first ERPO bill in 1999,^5^ adoption has expanded. As of February 2025, 21 states and the District of Columbia had implemented ERPO laws.^6^ However, robust evidence of their association with suicide prevention specifically remains limited and mixed.^6^

Prior evaluations have used 3 primary methods, ad hoc estimation, synthetic control methods, and difference in differences (DID), yielding mixed results. Early work used ad hoc methods but lacked sufficient data and power to achieve statistically significant estimates.^7,8,9^ Work that followed used synthetic control methods.^10,11,12,13^ However, the construction of synthetic control groups often did not explicitly consider the presence of all other potentially confounding non-ERPO firearm laws^10,12^ (or only partially considered such laws)^13^ that were passed simultaneously with or shortly after ERPO laws.^14^ For example, when not accounting for firearm laws passed at the same time or after the time of ERPO laws that might alter firearm availability and, in turn, suicide rates, estimation of the association between ERPO laws and suicide may be biased since the measured effect of ERPO laws may have inadvertently also captured the impact of non-ERPO firearm laws.^10,12^ Only 1 synthetic control study included a fully appropriate control group (with no important variation in the set of state-level firearm laws across counties within the state) but found no impact on suicide.^11^ The third line of previous work used DID,^13,15,16^ but these efforts were also susceptible to bias^17^ due to potential violations of the 2 primary DID assumptions: common-shocks and parallel-trends assumptions.^16^ Past DID research either did not consider^15^ or only partially considered^13^ the common-shocks assumption regarding non-ERPO firearm laws, meaning that the passage of new non-ERPO firearms laws must not be different across treatment and comparison groups. The parallel-trends assumption (preintervention suicide trends across the treatment and comparison groups must not be statistically different) was also not always considered.^15^ One well-controlled study^13^ did find that ERPOs reduced suicides when a broad group of petitioners was permitted (including law enforcement, family members, and clinicians). However, it assessed only overall suicides, leaving open the question of whether and to what extent individuals switched to other suicide methods after the passage of ERPO legislation.

In this study, we extend prior work by applying a more rigorous DID framework that simultaneously accounts for staggered treatment timing, treatment heterogeneity, and potentially confounding new firearm policies. First, we shift the unit of analysis used in most studies from the state to the county, aligning with how ERPOs are typically implemented and allowing us to control for local economic, health system, and social contexts. Second, we apply 2-way fixed-effects (TWFE) DID analyses that more fully account for factors that may bias results, including staggered implementation and treatment heterogeneity, while simultaneously addressing the common-shocks and parallel-trends assumptions.^18^

We focus on 4 states that passed ERPO laws without passing any other firearm policies at the same time as ERPO law passage and that also passed no firearm legislation for at least 1 year after ERPO law passage. We excluded from consideration 8 states where ERPO laws were passed at the same time as other firearm laws, since this situation makes it impossible to isolate the association between any particular new firearm policy and suicide outcomes, except in exceptional situations not present here. We also excluded 3 states that passed ERPO laws without passing other firearm legislation at the same time, but then immediately passed non-ERPO firearm legislation in the following year, since again, this situation also makes it impossible to determine the association between suicides and the ERPO law passed the year prior relative to the new firearm policy passed, except for any outcomes that may have happened in the same year the ERPO law was passed.

Our primary hypothesis was that, with other factors being equal, ERPO laws would reduce measurable firearm suicide mortality. Our secondary hypothesis was that ERPO laws would not increase measurable nonfirearm suicides, addressing a key concern in lethal means restriction: whether individuals substitute firearms with other suicide methods when firearm access is removed.

## Methods

### Data

This cohort study followed the Strengthening the Reporting of Observational Studies in Epidemiology (STROBE) reporting guideline. Institutional board review was not required because, in accordance with federal regulations, this project did not constitute human participant research as defined under 21 CFR 50.3 and under 45 CFR 46.102 per the policy of the Committee for Protection of Human Subjects of the University of California, Berkeley.

All data are at the US county level, and only complete cases were used. Our dependent variables were extracted using data from the Centers for Disease Control and Prevention Wide-Ranging Online Data for Epidemiologic Research for 2012 through 2022.^19^ Data were analyzed between February 6 and October 9, 2025. We conformed to the Centers for Disease Control and Prevention requirement of not reporting any statistics based on fewer than 10 overall cases.^20^ We computed 2 county-level annual mortality rates per 100 000 population: (1) firearm suicides (*International Statistical Classification of Diseases and Related Health Problems, Tenth Revision* codes X72 to X74) and (2) nonfirearm suicides (codes U03, X60 to X71, X75 to X84, and Y87.0). We chose the county as the unit of analysis rather than the state because ERPOs are usually issued by legal systems organized by county, and enforcement and implementation may vary by county. We identified 7 potential treatment states, Colorado, Massachusetts, Maryland, New Jersey, New Mexico, Oregon, and Rhode Island, based on the following criterion: an ERPO law passed within the 2018 to 2020 window. This criterion was designed to make it possible to identify a set of comparison states with at least 2 years of preperiod (2016 and 2017) data and at least 2 years of postperiod data (2021 and 2022) during which no laws regulating firearms were passed (2016 to 2022) according to the RAND firearm policy database (eAppendix in Supplement 1).^14^ This ensures that the set of comparison states would provide a clean comparison and that firearm law changes only occurred in treatment states. There were 8 states with no existing ERPO laws that passed no firearm laws of any kind from 2016 to 2022. This set of states became our comparison group: Alabama, Alaska, Michigan, Minnesota, Nebraska, North Carolina, Pennsylvania, and South Carolina. A detailed list of excluded states and the firearm policies enacted concurrently with ERPOs appears in eTable 1 in Supplement 1. A detailed list of states that passed ERPO laws alone and passed firearm laws 1 year after the ERPO laws were passed appears in eTable 2 in Supplement 1. Finally, the texts of all ERPO laws in all potential treatment states are listed in eTable 3 in Supplement 1.

Among the 7 potential treatment states that passed ERPO laws without passing other firearm laws at the same time, 3 (Colorado, Maryland, and Oregon) passed other firearm laws in the year immediately following ERPO law passage, making it difficult to separate the association between suicide and the ERPO laws from that between suicide and the newly passed non-ERPO firearm laws. This left 4 states that had at least 1 year after the passage of their ERPO laws during which no additional firearm laws were passed. We thus included 4 treatment states (Massachusetts, New Jersey, New Mexico, and Rhode Island) where no other firearm laws were passed the same year or the year after the ERPO laws were passed. If we assume no change in the associations of previously passed non-ERPO firearm laws, this should give us a consistent estimate of the ERPO associations.

#### Covariates

We included 4 sets of time-varying (2012 to 2022) county-level variables that may have confounded ERPO associations across time and within and across counties. These account for differences in the economic, legal administrative, mental health system, and social contexts.

The economic context included the percentage of the population in poverty, per capita income, and the employment rate, all of which have been associated with firearm mortality.^21,22,23^ Poverty rates came from the Small Area Income and Poverty Estimates program of the US Census,^24^ the employment rate from the Bureau of Labor Statistics,^25^ and per capita personal income from the Bureau of Economic Analysis.^26^

The legal administrative environment included the primary group responsible for enforcing ERPOs: police employees. Data on police employees came from the Federal Bureau of Investigation Crime Data Explorer.^27^

The mental health system context included the percentage of the population that was uninsured, psychiatrists per 100 000 population, Federally Qualified Health Centers (FQHC) per 100 000 population, Community Mental Health Centers (CMHC) per 100 000 population, and short-term general hospital beds per 100 000 population, most of which have been associated with suicide.^28,29,30,31,32^ The percentage of the population that was uninsured came from the Small Area Health Insurance Estimate program of the US Census,^33^ and psychiatrists, FQHCs, CMHCs, and short-term hospital beds per 100 000 population came from the Area Health Resources File maintained by the Health Resources and Services Administration.^34^

The social context included measures of nonsuicide deaths per 100 000 population, since the death of a loved one may trigger suicidal ideation.^35^ This measure includes deaths from COVID-19. In addition, alcohol availability, a factor in many suicides,^36^ was included: employees per 100 000 population in businesses that supply alcohol on site (North American Industry Classification System [NAICS] code 722511 [restaurants—full service] and NAICS code 722410 [drinking places—alcoholic beverage]) and employees per 100 000 population in businesses that supply alcohol for off-site consumption (NAICS code 445120 [convenience stores], NAICS code 445310 [beer, wine, and liquor stores], and NAICS code 447110 [gasoline stations with convenience stores]). All social context measures were derived from County Business Patterns maintained by the US Census.^37^

### Study Design

We studied the passage of ERPO laws as intention to treat rather than as treated. In other words, we evaluated the association of suicide with the legislative passage of ERPO laws and did not consider the extent to which the laws were effectively implemented. As-treated analysis is important but is outside the scope of this study. We used a quasi-experimental design: TWFE DID models^38,39^ to evaluate the associations between ERPO laws and firearm and nonfirearm suicides. This approach accounts for staggered implementation across treatment states and adjusts for both time and cohort heterogeneity as well as adding unbalanced panel corrections. Our TWFE DID model relied on 3 key assumptions.^40^

First, the common-shocks assumption requires that, apart from the ERPO law enacted in the treatment group, any other contemporaneous and future laws affect treatment and comparison groups similarly.^16,40^ Violations of the common-shocks assumption may result in effects being overstated or understated, depending on the context, which must be carefully considered.^17^ Overstating or understating the effect of ERPO laws may occur if any comparison states passed firearm laws at the same time or shortly after the time ERPO laws were passed in treatment states. To detect any deviations from the common-shocks assumption, we reviewed all firearm legislation changes in both the treatment and comparison groups.^14^ Our comparison group included all 8 states that neither had ERPO laws in place nor passed any firearm legislation from 2016 to 2022. Our treatment group satisfied the common-shocks assumption by including only states where no firearm laws were passed in the treatment states or comparison states during the year the ERPO laws were passed in the treatment states or within 1 year after.

Second, the parallel-trends assumption states that in the absence of the ERPO policy, outcomes would have followed their preintervention trends.^16,40^ Violations of the parallel-trends assumption make it impossible to attribute changes in outcomes to the intervention.

Third, the stable unit treatment value assumption assumes no spillover associations from treatment to comparison counties.^40^ While this assumption cannot easily be evaluated, the only consequence of its violation is that the estimated treatment effects will be attenuated, making our results conservative.

### Statistical Analysis

We implemented the estimation of TWFE DID models using the Mundlak TWFE estimator,^38,39,41^ which allowed us to account for staggered policy implementation and flexible modeling of unit-level heterogeneity. Models included county and year fixed effects. All counties were weighted by population to account for their relative contributions to the overall average treatment effect on the treated (ATT). We estimated dynamic ATTs, which represent the average association with the policy and how it evolves over time by calculating the mean of each staggered effect and comparing it with never-treated states. County-level fixed effects controlled for all time-invariant characteristics, such as baseline demographics, baseline political culture, baseline built environment, baseline health system infrastructure, and historical suicide and historical firearm trends. Therefore, any county-level characteristics that did not change over time or changed very little over time were effectively controlled. Year fixed effects control for national-level shocks (eg, COVID-19) or yearly trends that affect all counties in a given year, including federal policy changes, macroeconomic cycles, national economic changes, and national sociocultural changes. All analyses were conducted using Stata version 19.5 (StataCorp) of counties with complete data. We formally examined the parallel-trends assumption using event study analyses that account for staggered implementation and heterogeneity.^41^ Type 1 statistical significance was set at *P* ≤ .05 and all statistical significance tests are 2-sided. In addition, we used these same event study analyses to isolate the period during which our models adhere to the common-shocks assumption, which only extends to 1 year after ERPO passage for all states in the treatment group. In the second year after ERPO passage, Rhode Island implemented additional firearm laws, and in the third year after ERPO passage, all other treatment states (Massachusetts, New Jersey, and New Mexico) implemented additional firearm laws, which contaminate any estimates associated with ERPO law passage. We are thus limited to the intervention period and 1 additional year as periods during which the common-shocks assumption is valid.

## Results

Descriptive statistics are reported in Table 1, comparing counties in our treatment states (Massachusetts, New Jersey, New Mexico, and Rhode Island) with those in the 8 comparison states (Alabama, Alaska, Michigan, Minnesota, Nebraska, North Carolina, Pennsylvania, and South Carolina). In terms of economics, treatment counties had mean (SD) per capita income of $64 965 ($16 479), mean (SD) poverty rates of 10.6% (4.0%), and mean (SD) employment rates of 94.3% (2.3%) compared with comparison counties that had a mean (SD) per capita income of $48 781 ($12 747), mean (SD) poverty rates of 14% (5.3%), and mean (SD) employment rates of 94.5% (2.2%). Regarding administrative resources, treatment counties had police employees of mean (SD) 3.5 (2.4) per 100 000 and comparison counties had 2.9 (1.7) police employees per 100 000. Regarding health resources, treatment counties had a mean (SD) of 7.3% (4.4%) uninsured, 20.7 (15.8) psychiatrists per 100 000, 0.1 (0.2) CMHCs per 100 000, and 1.6 (1.5) FQHCs per 100 000, while comparison counties had 10.3% (4.2%) uninsured, 10.6 (11.7) psychiatrists per 100 000, 0.1 (0.4) CMHCs per 100 000, and 2.5 (3.7) FQHCs per 100 000. Regarding social factors, treatment counties had a mean (SD) of 1699 (625.7) employees per 100 000 in on-site consumer alcohol sales, 255 (91.5) employees per 100 000 in off-site consumer alcohol sales, and 775 (152.1) per 100 000 nonsuicide deaths. Comparison counties had a mean (SD) of 1633.6 (668) employees per 100 000 in on-site consumer alcohol sales and 342 (156.6) employees per 100 000 in off-site consumer alcohol sales, and 879.6 (216.8) per 100 000 nonsuicide deaths. Note that as long as our models do not violate the parallel-trends assumption, any differences between the treatment and comparison groups do not affect our findings.

**Table 1.** Descriptive Statistics^a^

Characteristic	Mean (SD)		
	Comparison group (n = 4962)	Treatment group (n = 1599)	Total (N = 6561)
Firearm and suicide			
Firearm suicides per 100 000 population	8.3 (4.5)	2.1 (1.2)	6.8 (4.8)
Nonfirearm suicides per 100 000 population	6.6 (3.3)	6.7 (1.8)	6.6 (3.0)
Economic environment			
Per capita income, $	48 781 (12 747)	64 965 (16 479)	52 726 (15 402)
Poverty, %	14.0 (5.3)	10.6 (4.0)	13.2 (5.2)
Employment, %	94.5 (2.2)	94.3 (2.3)	94.4 (2.2)
ERPO administrative resources			
Police employees per 100 000 population	2.9 (1.7)	3.5 (2.4)	3.0 (1.9)
Mental health resources			
Uninsured, %	10.3 (4.2)	7.3 (4.4)	9.6 (4.5)
Psychiatrists per 100 000 population	10.6 (11.7)	20.7 (15.8)	13.1 (13.5)
Federally Qualified Health Centers per 100 000 population	2.5 (3.7)	1.6 (1.5)	2.3 (3.3)
Community Mental Health Centers per 100 000 population	0.1 (0.4)	0.1 (0.2)	0.1 (0.4)
Short-term general hospital beds per 100 000 population	249.0 (161.4)	212.7 (101.2)	240.1 (149.8)
Social influences			
Nonsuicide deaths per 100 000 population	879.6 (216.8)	775.0 (152.1)	854.1 (207.8)
Employees per 100 000 population in consumer alcohol sales (on-site consumption)	1633.6 (668.0)	1699.0 (625.7)	1649.6 (658.4)
Employees per 100 000 population in consumer alcohol sales (off-site consumption)	342.0 (156.6)	255.0 (91.5)	320.8 (148.3)

Abbreviation: ERPO, Extreme Risk Protection Order.

^a^ Data are weighted by county population. Treatment group states were Massachusetts (ERPO law passed in 2018), New Jersey (2019), New Mexico (2020), and Rhode Island (2018). Comparison group states were Alabama, Alaska, Michigan, Minnesota, Nebraska, North Carolina, Pennsylvania, and South Carolina (no ERPO laws and no other firearm laws passed from 2016 to 2022).

Prepolicy trend tests were unable to reject the null hypothesis of parallel trends. No statistically significant differences in pretreatment trends were detected between treatment and comparison counties for firearm suicide rates (χ^2^ = 0.08; *P* = .99) or nonfirearm suicide rates (χ^2^ = 3.69; *P* = .16) (Table 2). The information in Table 2 is depicted visually in eFigure 1 and eFigure 2 in Supplement 1.

**Table 2.** Results of Passage of ERPO Laws in Massachusetts, New Jersey, New Mexico, and Rhode Island^a^

Period	Firearm suicides		Nonfirearm suicides	
	Per 100 000 (95% CI)	*P* value	Per 100 000 (95% CI)	*P* value
Dynamic ATT				
3 y Prior	−0.14 (−1.69 to 1.40)	.86	−2.68 (−5.42 to 0.07)	.06
2 y Prior	−0.13 (−1.29 to 1.03)	.82	−1.16 (−2.71 to 0.39)	.14
1 y Prior	[Reference]	NA	[Reference]	NA
Intervention period	−0.14 (−1.24 to 0.96)	.80	0.41 (−1.12 to 1.94)	.60
1 y After	−3.79 (−6.74 to −0.83)	.01	−2.45 (−6.84 to 1.93)	.27
2 y After	−0.40 (−6.12 to 5.31)	.89	1.93 (−10.30 to 14.17)	.76
3 y After	−0.89 (−3.94 to 2.16)	.57	2.46 (−1.74 to 6.66)	.25
4 y After	−2.47 (−5.78 to 0.85)	.15	8.08 (3.03 to 13.14)	.002
Parallel-trends test (*P* value)	.99	NA	.16	NA
Observations, No.	6561	NA	6561	NA

Abbreviations: ATT, average treatment effect on the treated; ERPO, Extreme Risk Protection Order; NA, not applicable.

^a^ Data are weighted by county population. Treatment group states were Massachusetts (ERPO law passed in 2018), New Jersey (2019), New Mexico (2020), and Rhode Island (2018). Comparison group states were Alabama, Alaska, Michigan, Minnesota, Nebraska, North Carolina, Pennsylvania, and South Carolina (no ERPO laws and no other firearm laws passed from 2016 to 2022).

Table 2 reports the main DID dynamic ATT event study results. We found no association of ERPO laws with suicide in the year they were passed (−0.14; 95% CI, −1.24 to 0.96; *P* = .80), but an association was found in the first year after they were passed, with 3.79 fewer firearm suicides per 100 000 population occurring (95% CI, −6.74 to −0.83; *P* = .01). This corresponds to an estimated 675 firearm suicide deaths prevented across the treatment period, calculated as 3.79 × (17.8 million/100 000), where 17.8 million represents the treatment population. With regard to nonfirearm suicides, we did not find a statistically significant association in the year ERPO laws were passed (0.41; 95% CI, −1.21 to 1.94; *P* = .60) or in the next year (−2.45; 95% CI, −6.84 to 1.93; *P* = .27) (Table 2).

## Discussion

We hypothesized that ERPOs would be associated with measurable reductions in firearm suicides without corresponding measurable increases in nonfirearm suicides. Our findings are unable to reject either hypothesis. The ERPO laws passed in Massachusetts, New Jersey, New Mexico, and Rhode Island were associated with a mean county-level reduction of 3.79 suicides per 100 000 population in firearm suicide rates, with no evidence of measurable suicide method substitution. To our knowledge, this is the first study to evaluate ERPO laws’ impacts in terms of firearm suicides using county-level data and accounting for staggered implementation, heterogeneity, the key assumptions of the DID model, including parallel trends, and all state firearm policy confounding from other state-level firearm legislation that might bias the measurement of ERPO impacts. Our model allowed us to control for county baseline variation and characterize important time-varying economic, health system, and social factors.

Note that we excluded states other than Massachusetts, New Jersey, New Mexico, and Rhode Island only to satisfy the assumptions required for valid DID estimation; this in no way implies a lack of effectiveness of ERPO laws in other states. It only implies that we were unable to assess their association with suicide rigorously.

These findings have policy relevance. Our results suggest that the continued adoption of ERPO laws would further stem the tide of firearm suicides without a measurable compensatory effect on nonfirearm suicides. Future research should examine the mechanisms of implementation that enhance the ERPO impact in firearm suicide prevention and whether any disparities exist across populations by socioeconomic status.

### Limitations

This study has some limitations. Our choice of comparison states and their counties was limited to meet the methodologic requirements of our DID approach. Given this, the external validity of the study may be limited to these states.

## Conclusions

This cohort study provides timely and policy-relevant evidence that ERPO laws, when well implemented, can serve as effective public health tools to reduce firearm suicides. Our study conservatively estimated that ERPO laws prevented 675 firearm suicide deaths during the treatment period. The absence of significantly increased nonfirearm suicides further supports ERPOs as targeted interventions that reduce access to lethal means without driving individuals toward alternative methods. We encourage other states to implement similar laws to those enacted in Massachusetts, New Jersey, New Mexico, and Rhode Island that protect individuals who are at the highest risk of suicide by firearms.

## Notes

Supplement 1.
eAppendix
**eTable 1.** States With ERPO Laws During 2018-2020 Excluded Due to Passing Other Firearm Laws During the Same Year **eTable 2.** States Passing ERPO Laws Alone That Had Firearm Laws Passed Within 1 Year After the Passage of an ERPO Law **eTable 3.** Extreme Risk Protection Order (ERPO) Laws of Potential Treatment States **eFigure 1.** Vertical Bar Graph of Firearm Suicide Event Study Analyses **eFigure 2.** Vertical Bar Graph of Nonfirearm Suicide Event Study Analyses

Supplement 2.
Data Sharing Statement
