# Supplementary material for: Extreme Risk Protection Orders and Firearm and Nonfirearm Suicides in the US
Source: JAMA Health Forum. 2026 Jan 30;7(1):e256442. doi: 10.1001/jamahealthforum.2025.6442 (PMC12859719; doi:10.1001/jamahealthforum.2025.6442)
Supplement: Supplement 1. — eAppendix eTable 1. States With ERPO Laws During 2018-2020 Excluded Due to Passing Other Firearm Laws During the Same Year eTable 2. States Passing ERPO Laws Alone That Had Firearm Laws Passed Within 1 Year After the Passage of an ERPO Law eTable 3. Extreme Risk Protection Order (ERPO) Laws of Potential Treatment States eFigure 1. Vertical Bar Graph of Firearm Suicide Event Study Analyses eFigure 2. Vertical Bar Graph of Nonfirearm Suicide Event Study Analyses [file jamahealthforum-e256442-s001.pdf]

Supplemental Online Content

Brown TT, Kaplan MS, Yan Z, Xiao Y. Extreme Risk Protection Orders and firearm and nonfirearm suicides in the US. *JAMA Health Forum*. 2026;7(1):e256442. doi:10.1001/jamahealthforum.2025.6442

eAppendix

eTable 1. States With ERPO Laws During 2018-2020 Excluded Due to Passing Other Firearm Laws During the Same Year

eTable 2. States Passing ERPO Laws Alone That Had Firearm Laws Passed Within 1 Year After the Passage of an ERPO Law

eTable 3. Extreme Risk Protection Order (ERPO) Laws of Potential Treatment States

eFigure 1. Vertical Bar Graph of Firearm Suicide Event Study Analyses

eFigure 2. Vertical Bar Graph of Nonfirearm Suicide Event Study Analyses

© 2025 Brown TT et al. *JAMA Health Forum*.

eAppendix

Based on RAND firearm policy data,[1] we examined state ERPO laws passed within a 3-year period (2018-2020) in order to give us at least two-years before and two-years after the passage of an ERPO law (2016-2022) to determine parallel trends and outcomes. This required a set of comparison states without ERPO laws that also did not pass any firearm legislation during the period 2016-2022. During this period 15 states passed an ERPO law. To avoid confounding of the effect of ERPOs with other firearm laws in this study, we excluded 8 states whose ERPO laws were put in place at the same time as another firearm law. See eTable 1 for a list of these states and the categories of the other firearms laws passed at the same time. These states were excluded because simultaneous passage of multiple firearm laws makes it impossible, except in very special cases, to disentangle the effects of any particular firearms law on firearm suicide.

[1] Hoch E, Nabel A, Morral AR, Schell TL, Smucker S. *Development of the RAND State Firearm Law Database and Supporting Materials*.; 2025. Accessed May 1, 2025. https://www.rand.org/pubs/tools/TLA243-2-v3.html

© 2025 Brown TT et al. *JAMA Health Forum*.

eTable 1. **States with ERPO Laws During 2018-2020 Excluded Due to Passing Other Firearm Laws During the Same Year**

State	Year of ERPO Law	Firearm Laws Passed During the Same Year
Delaware	2018	Mental Health Restrictions
Florida	2018	Background Checks<br>Mental Health Restrictions Prohibiting Possession
Hawaii	2020	Ghost Gun Restrictions
Illinois	2019	Certification of Gun Dealers
Nevada	2020	Background Checks
New York	2019	Background Checks<br>Child Access Restrictions
Vermont	2018	Background Checks<br>Minimum Age Restrictions<br>Domestic Violence Arrest<br>Large Capacity Magazine Restriction
Virginia	2020	Protective Order<br>Background Checks<br>Number of Guns Purchased Restrictions<br>Reporting Lost/Stolen Gun

© 2025 Brown TT et al. *JAMA Health Forum*.

eTable 2. **States Passing ERPO Laws Alone that had Firearm Laws Passed Within One Year After the Passage of an ERPO Law.**

State	Year of ERPO Law	Firearm Laws Passed One Year After ERPO Law (Year)
Colorado	2019	Expanded ERPO (2020)<br><br>Required Reporting of Lost/Stolen Firearm (2021)
Maryland	2018	Gun Trafficking Restrictions Expanded (2019)
Oregon	2018	Domestic Violence Firearm Removal Expanded (2019)

© 2025 Brown TT et al. *JAMA Health Forum*.

eTable 3. Extreme Risk Protection Order (ERPO) Laws of Potential Treatment States

Colorado (2019)

CO § 13-14.5-104	13-14.5-104. Petition for extreme risk protection order. (1) A petition for an extreme risk protection order may be filed by a family or household member of the respondent or a law enforcement officer or agency. If the petition is filed by a law enforcement officer or agency, the officer or agency shall be represented in any judicial proceeding by a county or city attorney upon request. If the petition is filed by a family or household member, the petitioner, to the best of his or her ability, shall notify the law enforcement agency in the jurisdiction where the respondent resides of the petition and the hearing date with enough advance notice to allow for participation or attendance. Upon the filing of a petition, the court shall appoint an attorney to represent the respondent, and the court shall include the appointment in the notice of hearing provided to the respondent pursuant to section 13-14.5-105 (1)(a). The respondent may replace the attorney with an attorney of the respondent's own selection at anytime at the respondent's own expense. Attorney fees for the attorney appointed for the respondent shall be paid by the court.
CO § 13-14.5-103	Temporary extreme risk protection orders. (1) A family or household member of the respondent or a law enforcement officer or agency may request a temporary extreme risk protection order without notice to the respondent by including in the petition for an extreme risk protection order an affidavit, signed under oath and penalty of perjury, supporting the issuance of a temporary extreme risk protection order that sets forth the facts tending to establish the grounds of the petition or the reason for believing they exist. The petition shall comply with the requirements of section 13-14.5-104 (3). If the petitioner is a law enforcement officer or law enforcement agency, the law enforcement officer or law enforcement agency shall concurrently file a sworn affidavit for a search warrant pursuant to section 16-3-301.5 to search for any firearms in the possession or control of the respondent at a location or locations to be named in the warrant. If a petition pursuant to section 27-65-106 is also filed against the respondent, the court can hear that petition at the same time as the hearing for a temporary extreme risk protection order or the hearing for a continuing extreme risk protection order.<br><br>(2) in considering whether to issue a temporary extreme risk protection order pursuant to this section, the court shall consider all relevant evidence, including the evidence described in section 13-14.5-105 (3). If a court finds by a preponderance of the evidence that, based on the evidence presented pursuant to section 13-14.5-105 (3), the respondent poses a significant risk of causing personal injury to self or others in the near future by having in his or her custody or control a firearm or by purchasing, possessing, or receiving a firearm, the court shall issue a temporary extreme risk protection order.

Massachusetts (2018)

Mass. Gen. Laws ch. 140, § 121; Mass. Gen. Laws ch. 140, § 129B(C); Mass. Gen. Laws ch. 140, § 131(C); Mass. Gen. Laws ch. 140, §131R-Z;	CHAPTER 209E<br>EXTREME RISK PROTECTIVE ORDERS<br><br>Section 1. As used in this chapter, the following terms shall have the following meanings, unless the context clearly requires otherwise:-<br>“Court”, the superior, district, or Boston municipal court departments of the trial court.<br>“Extreme risk protective order”, an order in writing, issued and signed by the court pursuant to section 6, prohibiting and enjoining a named person from having in his or her custody or control, owning, purchasing, possessing, or receiving any firearms or ammunition for a period of 1 year.<br>“Family or household member”, a person who:<br>(a) is or was married to the respondent;<br>(b) is or was residing with the respondent in the same household;<br>(c) is or was related by blood or marriage to the respondent;<br>(d) has or is having a child in common with the respondent, regardless of whether they have ever married or lived together; or<br>(e) is or has been in a substantive dating or engagement relationship with the respondent, which shall be adjudged by district or Boston municipal courts consideration of the following factors: (1) the length of time of the relationship; (2) the type of relationship; (3) the frequency of interaction between the parties; and (4) if the relationship has been terminated by either person, the length of time elapsed since the termination of the relationship.<br>“Health care provider”, a licensed physician, licensed clinical psychologist, licensed social worker or licensed mental health clinician.<br>“Petitioner”, a family or household member, health care provider, or law enforcement officer or district attorney with jurisdiction over the respondent’s residence, who files a petition requesting a temporary or 1-year extreme risk protective order.<br>''Protective order issued by another jurisdiction'', any injunction or other order issued by a court of another state, territory or possession of the United States, the Commonwealth of Puerto Rico, or the District of Columbia, or tribal court that is issued for the purpose of prohibiting a person from having in his or her custody or control, owning, purchasing, possessing, or receiving any firearms or ammunition because that person poses a significant danger of causing physical harm to themselves, the petitioner, or anyone else by having in his or her custody or control, owning, purchasing, possessing, or receiving such firearm or ammunition.<br>“Respondent”, the person identified in the petition filed pursuant to this chapter, against whom an order is sought.<br>“Temporary extreme risk protective order”, a written order issued pursuant to section 8, prohibiting and enjoining a named person from having in his or her custody or control, owning, purchasing, possessing, or receiving any

© 2025 Brown TT et al. *JAMA Health Forum*.

firearms or ammunition for a period of 10 days, prior to a hearing for a 1-year extreme risk protective order.

Section 2. Proceedings under this chapter shall be filed, heard and determined in the superior court department or respective divisions of the district court departments or the Boston municipal court department having jurisdiction over the respondent's residence.

Section 3. The administrative justices of the superior court, district court and the Boston municipal court departments shall jointly promulgate a form of petition for use under this chapter which shall be in such form and language to permit a person to prepare and file such petition pro se.

Section 4. Upon the filing of a petition pursuant to this chapter, a petitioner shall be informed that the proceedings hereunder are civil in nature and that violations of orders issued hereunder are criminal in nature. No filing fee shall be charged for the filing of the petitioner. Neither the petitioner nor the petitioner's attorney shall be charged for certified copies of any orders entered by the court, or any copies of the file reasonably required for future court action or as a result of the loss or destruction of petitioner's copies.

Section 5. (a) A family or household member, health care provider, or a law enforcement officer or district attorney with jurisdiction over the respondent's residence, may seek an extreme risk protective order by filing a verified petition on a form approved pursuant to section 3 in the superior, district, or the Boston municipal court department having jurisdiction over the respondent's residence.
A healthcare provider may seek an extreme risk protective order or a temporary extreme risk protective order against a patient pursuant to this chapter when: (i) the patient has communicated to the healthcare provider an explicit threat to kill or inflict serious bodily injury upon a reasonably identified victim or victims, the patient has the apparent intent and ability to carry out the threat, and the health care provider knows or has reason to believe that the patient controls, owns or possesses a firearm, and (ii) the patient has a history of physical violence which is known to the healthcare provider, the healthcare provider has a reasonable basis to believe that there is a clear and present danger that the patient will attempt to kill or inflict serious bodily injury against a reasonably identified victim or victims, and the health care provider knows or has reason to believe that the patient controls, owns or possesses a firearm. Healthcare providers shall otherwise be bound by federal law and regulations relative to the confidentiality of patient health information.
(b) The petition shall set forth the grounds for issuance of the order and shall describe the number, types, and locations of any firearms or ammunition presently believed by the petitioner to be owned, possessed or controlled by the respondent. The petition shall also state whether there is an existing abuse prevention order pursuant to chapter 209A in effect

© 2025 Brown TT et al. *JAMA Health Forum*.

governing the respondent, and whether there is any pending lawsuit, complaint, petition, or other action between the parties under the laws of this state. The court administrator shall verify the terms of any existing order governing the parties. The court may not delay granting relief because of the existence of a pending action between the parties or the necessity of verifying the terms of an existing order. An extreme risk protective order may be granted whether or not there is a pending action between the parties.
(c) Upon receipt of the petition, the court shall set a date for a hearing within 10 days, regardless of whether the court issues a temporary extreme risk protective order pursuant to section 8. Written notice of the hearing shall be personally served on the respondent by a law enforcement officer, or if personal service by a law enforcement officer is not possible, in accordance with the laws relative to service of process in the commonwealth. If the court does issue a temporary extreme risk protective order pursuant to section 8, notice of the hearing shall be served on the respondent with the temporary order. Written notice of the hearing shall be sent to the petitioner by certified mail.

Section 6. (a) A petitioner requesting an extreme risk protective order shall include in the petition detailed allegations based on personal knowledge that the respondent poses a significant danger of causing physical harm to themselves, the petitioner, or others by having in his or her custody or control, owning, purchasing, possessing, or receiving a firearm or ammunition.
(b) Before a hearing for an extreme risk protective order, the court shall:
(i) ensure that a reasonable search has been conducted of all available records to determine whether the respondent owns any firearms or ammunition, including confirmation from the local licensing authority with jurisdiction over the respondent of the firearms owned or possessed by the respondent; and
(i) ensure that a reasonable search has been conducted for criminal history records related to the respondent.
(c) In determining whether to issue an extreme risk protective order under this section, the court shall consider all relevant evidence presented by the petitioner, including an examination under oath of the petitioner and any witnesses the petitioner may produce, and may also consider other relevant evidence relative to the respondent, including, but not limited to:
(i) unlawful, reckless or negligent use, display, storage, possession or brandishing of a firearm;
(ii) acts or threats of violence against oneself or another, whether or not this violence involves a firearm;
(iii) violation of a protective order issued pursuant to chapter 209A or a similar law in another state;
(iv) abuse of controlled substances or alcohol or any criminal offense that involves controlled substances or alcohol; or
(v) the recent acquisition of firearms, ammunition, or other deadly weapons.

© 2025 Brown TT et al. *JAMA Health Forum*.

	The court may also consider the time that has elapsed since the events described above.<br>The respondent shall have an opportunity to present evidence and witnesses at the respondent's discretion, to rebut the evidence presented by the petitioner. The court shall consider all relevant evidence presented by the respondent.<br>(d) If the court finds that the petitioner has proven by a preponderance of the evidence that the respondent poses a significant danger of physical harm to themselves, the petitioner, or anyone else by having in his or her custody or control, owning, purchasing, possessing, or receiving a firearm or ammunition, the court shall issue an extreme risk protective order, which shall prohibit the respondent from having in his or her custody or control, owning, possessing, purchasing, or receiving, or attempting to purchase or receive a firearm or ammunition while the order is in effect for a period of 1-year, unless terminated sooner pursuant to section 7.<br>(e) An extreme risk protective order issued pursuant to this section shall include all of the following:<br>(i) a statement that the respondent may not have in his or her custody or control, own, possess, purchase, or receive, or attempt to purchase or receive a firearm or ammunition while the order is in effect, which shall be for a period of 1 year unless otherwise terminated sooner;<br>(ii) a description of the requirements for relinquishment of firearms and ammunition pursuant to section 9;<br>(iii) a statement of the grounds supporting the issuance of the order;<br>(iv) the date and time the order expires;<br>(v) the address of the court that issued the order;<br>(vi) a statement that the respondent shall have the right to request 1 hearing to terminate the order at any time during its effective period; and<br>(vii) a statement that the respondent may seek the advice of an attorney as to any matter connected with the order.<br>(f) An extreme risk protective order issued pursuant to this section shall be personally served on the respondent with an order of suspension and surrender pursuant to section 9 by a law enforcement officer, or if personal service by a law enforcement officer is not possible, in accordance with state laws regarding service of process. §5–603
Ch. 140, §§ 121, 131R, 131S, 131T	M.G.L.A. 140 § 121 "Petitioner", the family or household member, or the licensing authority of the municipality where the respondent resides, filing a petition. §131R(a): A petitioner who believes that a person holding a license to carry firearms or a firearm identification card may pose a risk of causing bodily injury to self or others may, on a form furnished by the court and signed under the pains and penalties of perjury, file a petition in court. § 131S: Upon granting a petition, the court shall issue an extreme risk protection order and shall order the respondent to surrender any licenses to carry firearms, firearms identification cards and all firearms, rifles, shotguns, machine guns, weapons and ammunition which the respondent then controls, owns or possesses, to the licensing authority of the municipality where the respondent resides. The court shall enter written findings as to the basis of its order within 24 hours of granting the order.

© 2025 Brown TT et al. *JAMA Health Forum*.

The court may modify, suspend or terminate its order at any subsequent time upon motion by either party; provided, however, that due notice shall be given to the respondent and petitioner, and the court shall hold a hearing on said motion. When the petitioner's address is confidential to the respondent as provided in subsection (d) of section 131R and the respondent has filed a motion to modify the court's order, the court shall be responsible for notifying the petitioner. In no event shall the court disclose any such confidential address. § 131T: (a) Upon the filing of a petition pursuant to section 131R, the court may issue an emergency extreme risk protection order without notice to the respondent and prior to the hearing required pursuant to subsection (a) of section 131S if the court finds reasonable cause to conclude that the respondent poses a risk of causing bodily injury to the respondent's self or others by being in possession of a license to carry firearms or a firearm identification card or having in the respondent's control, ownership or possession a firearm, rifle, shotgun, machine gun, weapon or ammunition.

Upon issuance of an emergency extreme risk protection order pursuant to this section, the clerk magistrate of the court shall notify the respondent pursuant to subsection (e) of section 131S. An order issued under this subsection shall expire 10 days after its issuance unless a hearing is scheduled pursuant to subsection (a) or (b) of said section 131S or at the conclusion of a hearing held pursuant to said subsection (a) or (b) of said section 131S unless a permanent order is issued by the court pursuant to subsection (d) of said section 131S.

(b) When the court is closed for business, a justice of the court may grant an emergency extreme risk protection order if the court finds reasonable cause to conclude that the respondent poses a risk of causing bodily injury to the respondent's self or others by being in possession of a license to carry firearms or firearm identification card or by having in the respondent's control, ownership or possession of a firearm, rifle, shotgun, machine gun, weapon or ammunition. In the discretion of the justice, such relief may be granted and communicated by telephone to the licensing authority of the municipality where the respondent resides, which shall record such order on a form of order promulgated for such use by the chief justice of the trial court and shall deliver a copy of such order on the next court business day to the clerk-magistrate of the court. If relief has been granted without the filing of a petition pursuant to section 131R, the petitioner shall appear in court on the next available court business day to file a petition. An order issued under this subsection shall expire at the conclusion of the next court business day after the order was issued unless a petitioner has filed a petition with the court pursuant to said section 131R and the court has issued an emergency extreme risk protection order pursuant to subsection (a).

© 2025 Brown TT et al. *JAMA Health Forum*.

Maryland (2018)

Md. Code Ann., Pub. Safety §§ 5-601(E)(2); 5-602; 5-603	§5–603.<br>(a) (1) When a petition is filed with a District Court commissioner under § 5–602(b)(2) of this subtitle, the commissioner may enter an interim extreme risk protective order to prohibit the respondent from possessing a firearm if the commissioner finds that there are reasonable grounds to believe that the respondent poses an immediate and present danger of causing personal injury to the respondent, the petitioner, or another by possessing a firearm.<br>(2) In determining whether to enter an interim extreme risk protective order under this section, the commissioner shall consider:<br>(i) all relevant evidence presented by the petitioner; and<br>(ii) the amount of time that has elapsed since any of the events described in the petition.<br>(3) The interim extreme risk protective order shall:<br>(i) order the respondent to surrender to law enforcement authorities any firearm and ammunition in the respondent's possession; and<br>(ii) prohibit the respondent from purchasing or possessing any firearm or ammunition for the duration of the interim extreme risk protective order.<br>(4) If, based on the petition, the commissioner finds probable cause to believe that the respondent meets the requirements for emergency evaluation under Title 10, Subtitle 6 of the Health – General Article, the commissioner shall refer the respondent to law enforcement for a determination of whether the respondent should be taken for an emergency evaluation.<br>(b) (1) (i) An interim extreme risk protective order shall state the date, time, and location for a temporary extreme risk protective order hearing and a tentative date, time, and location for a final extreme risk protective order hearing.
Md. Code Ann., Pub. Safety §§ 5-601(E)(2); 5-602; 5-603	§5–601.<br>(a) In this subtitle the following words have the meanings indicated.<br>(b) "Ammunition" has the meaning stated in § 5–133.1 of this title.<br>(c) "Extreme risk protective order" means a civil interim, temporary, or final protective order issued in accordance with this subtitle.<br>(d) "Firearm" has the meaning stated in § 5–101 of this title.<br>(e) (1) "Petitioner" means an individual who files a petition for an extreme risk protective order under this subtitle.<br>(2) "Petitioner" includes:<br>(i) a physician, psychologist, clinical social worker, licensed clinical professional counselor, clinical nurse specialist in psychiatric and mental health nursing, psychiatric nurse practitioner, licensed clinical marriage or family therapist, or health officer or designee of a health officer who has examined the individual;<br>(ii) a law enforcement officer;<br>(iii) the spouse of the respondent;

© 2025 Brown TT et al. *JAMA Health Forum*.

(iv) a cohabitant of the respondent;
(v) a person related to the respondent by blood, marriage, or adoption;
(vi) an individual who has a child in common with the respondent;
(vii) a current dating or intimate partner of the respondent; or
(viii) a current or former legal guardian of the respondent.
(f) "Respondent" means a person against whom a petition for an extreme risk protective order is filed.
§5–602.
(a) (1) A petition for an extreme risk protective order shall:
(i) be signed and sworn to by the petitioner under the penalty of perjury;
(ii) include any information known to the petitioner that the respondent poses an immediate and present danger of causing personal injury to the respondent, the petitioner, or another by possessing a firearm;
(iii) set forth specific facts in support of the information described in item (ii) of this
paragraph;
§5–603(a)(1) When a petition is filed with a District Court commissioner under § 5-602(b)(2) of this subtitle, the commissioner may enter an interim extreme risk protective order to prohibit the respondent from possessing a firearm if the commissioner finds that there are reasonable grounds to believe that the respondent poses an immediate and present danger of causing personal injury to the respondent, the petitioner, or another by possessing a firearm.

New Jersey (2019)

N.J. Stat. Ann. §§ 2C:58-21, 23	NJ ST 2C:58–21 "Petitioner" means a family or household member or law enforcement officer. NJ ST 2C:58–23 a petitioner may file a petition, as prescribed by the Administrative Director of the Courts, for a temporary extreme risk protective order in the court in accordance with the Rules of Court alleging that the respondent poses a significant danger of bodily injury to self or others by having custody or control of, owning, possessing, purchasing, or receiving a firearm. The petition shall be heard by the court in an expedited manner.

New Mexico (2020)

NM ST § 40-17-5	A. A petition for an extreme risk firearm protection order shall be filed only by a law enforcement officer employed by a law enforcement agency; provided that, if the respondent is a law enforcement officer, the petition shall be filed by the district attorney or the attorney general.<br>B. A petitioner may file a petition with the court requesting an extreme risk firearm protection order that shall enjoin the respondent from having in the respondent's possession, custody or control any firearm and shall further

© 2025 Brown TT et al. *JAMA Health Forum*.

	enjoin the respondent from purchasing, receiving or attempting to purchase, possess or receive any firearm while the order is in effect.
NM ST § 40-17-5 (d)	D. A law enforcement officer shall file a petition for an extreme risk firearm protection order upon receipt of credible information from a reporting party that gives the agency or officer probable cause to believe that a respondent poses a significant danger of causing imminent personal injury to self or others by having in the respondent's custody or control or by purchasing, possessing or receiving a firearm.

Oregon (2018)

Or. Rev. Stat. Ann. §§ 166.525, 166.527	166.525 Definitions. As used in ORS 166.525 to 166.543:<br>(6) "Petitioner" means a person who petitions for an order under ORS 166.525 to 166.543.<br>(7) "Respondent" means a person against whom an order is filed under ORS 166.525 to 166.543. [2017 c.737 §1]<br><br>166.527 Petition for ex parte order; issuance and service of order; request for hearing. (1) A law enforcement officer or a family or household member of a person may file a petition requesting that the court issue an extreme risk protection order enjoining the person from having in the person's custody or control, owning, purchasing, possessing or receiving, or attempting to purchase or receive, a deadly weapon. (5)(a) The petitioner has the burden of proof at the ex parte hearing.

Rhode Island (2018)

R.I. Gen. Laws § 8-8.3-4	(a) A petition for an extreme risk protection order shall be filed only by a law enforcement agency.<br>(b) A petitioner may file a petition with the court requesting an extreme risk protection order that shall enjoin the respondent from having in their possession, custody or control any firearms and shall further enjoin the respondent from purchasing, receiving or attempting to purchase or receive any firearms while the order is in effect. The petitioner shall concurrently file a sworn affidavit for a search warrant pursuant to chapter 5 of title 12 for the search of any firearms in the possession, custody, or control of the respondent....8-8.3-4. Temporary orders -- Proceedings.<br><br>(a) Upon the filing of a petition under this chapter, the court may enter a temporary order if the court finds there is probable cause from specific facts shown by the petition that the respondent poses a significant danger of causing imminent personal injury to self or others by having in their custody or control, or by purchasing, possessing, or receiving, a firearm before notice can be served and a hearing held.

© 2025 Brown TT et al. *JAMA Health Forum*.

R.I. Gen. Laws § 8-8.3-4	(a) A petition for an extreme risk protection order shall be filed only by a law enforcement agency.<br>(b) A petitioner may file a petition with the court requesting an extreme risk protection order that shall enjoin the respondent from having in their possession, custody or control any firearms and shall further enjoin the respondent from purchasing, receiving or attempting to purchase or receive any firearms while the order is in effect. The petitioner shall concurrently file a sworn affidavit for a search warrant pursuant to chapter 5 of title 12 for the search of any firearms in the possession, custody, or control of the respondent....8-8.3-4. Temporary orders -- Proceedings.<br>(a) Upon the filing of a petition under this chapter, the court may enter a temporary order if the court finds there is probable cause from specific facts shown by the petition that the respondent poses a significant danger of causing imminent personal injury to self or others by having in their custody or control, or by purchasing, possessing, or receiving, a firearm before notice can be served and a hearing held.

© 2025 Brown TT et al. *JAMA Health Forum*.

eFigure 1. Vertical Bar Graph of Firearm Suicide Event Study Analyses

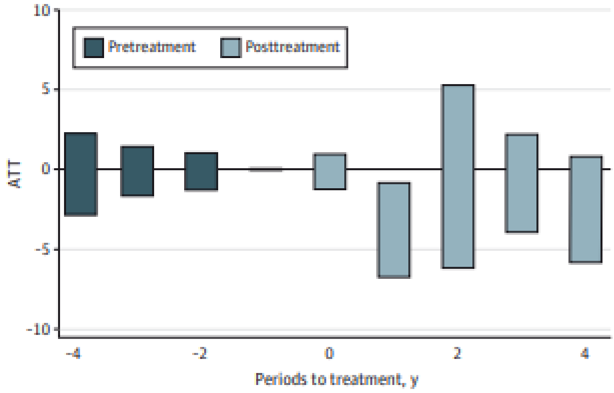

Firearm suicides per 100 000 population. ATT indicates average treatment effect on the treated.

© 2025 Brown TT et al. *JAMA Health Forum*.

eFigure 2. Vertical Bar Graph of Nonfirearm Suicide Event Study Analyses

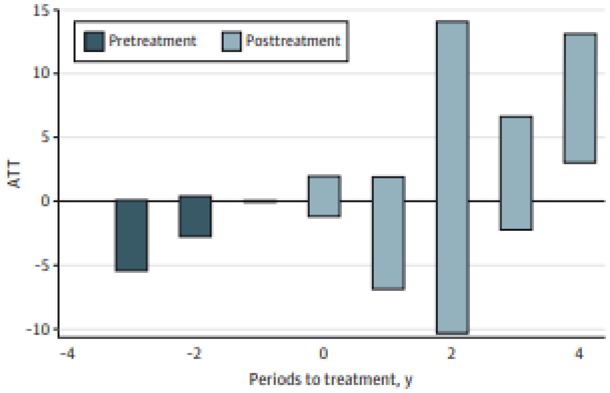

Nonfirearm suicides per 100 000 population. ATT indicates average treatment effect on the treated.

© 2025 Brown TT et al. *JAMA Health Forum*.
