# Supplementary material for: Extreme Risk Protection Orders and Firearm and Nonfirearm Suicides in the US
Source: JAMA Health Forum. 2026 Jan 30;7(1):e256442. doi: 10.1001/jamahealthforum.2025.6442 (PMC12859719; doi:10.1001/jamahealthforum.2025.6442)
Supplement: Supplement 2. — Data Sharing Statement [file jamahealthforum-e256442-s002.pdf]

Data Sharing Statement

Brown. Extreme Risk Protection Orders and Firearm and Nonfirearm Suicides in the US. *JAMA Health Forum*. Published January 30, 2026. doi:10.1001/jamahealthforum.2025.6442

Data
Data available: No

Additional Information
Explanation for why data not available: All data used in this study are publicly available.
